# Heterogeneity of mesenchymal stem cells in cartilage regeneration: from characterization to application

**DOI:** 10.1038/s41536-021-00122-6

**Published:** 2021-03-19

**Authors:** Kangkang Zha, Xu Li, Zhen Yang, Guangzhao Tian, Zhiqiang Sun, Xiang Sui, Yongjing Dai, Shuyun Liu, Quanyi Guo

**Affiliations:** 1 Medical School of Chinese PLA, Beijing, China; 2 Institute of Orthopaedics, Chinese PLA General Hospital, Beijing Key Lab of Regenerative Medicine in Orthopaedics, Key Laboratory of Musculoskeletal Trauma & War Injuries, PLA, Beijing, China; 3 School of Medicine, Nankai University, Tianjin, China; 4 Musculoskeletal Research Laboratory, Department of Orthopedics and Traumatology, Innovative Orthopaedic Biomaterial and Drug Translational Research Laboratory, Li Ka Shing Institute of Health Sciences, The Chinese University of Hong Kong, Hong Kong, China

**Keywords:** Mesenchymal stem cells, Trauma

## Abstract

Articular cartilage is susceptible to damage but hard to self-repair due to its avascular nature. Traditional treatment methods are not able to produce satisfactory effects. Mesenchymal stem cells (MSCs) have shown great promise in cartilage repair. However, the therapeutic effect of MSCs is often unstable partly due to their heterogeneity. Understanding the heterogeneity of MSCs and the potential of different types of MSCs for cartilage regeneration will facilitate the selection of superior MSCs for treating cartilage damage. This review provides an overview of the heterogeneity of MSCs at the donor, tissue source and cell immunophenotype levels, including their cytological properties, such as their ability for proliferation, chondrogenic differentiation and immunoregulation, as well as their current applications in cartilage regeneration. This information will improve the precision of MSC-based therapeutic strategies, thus maximizing the efficiency of articular cartilage repair.

## Introduction

Articular cartilage resides on the surface of musculoskeletal joints and is capable of withstanding complex mechanical stimuli, such as pressure and shear force. Owing to its lack of blood vessels, articular cartilage mainly receives required nutrients by synovial fluid infiltration, making it difficult for the cartilage to heal itself after injury^1^. Without proper treatment, cartilage lesions may result in post traumatic osteoarthritis, which can lead to joint pain, deformity, and movement disorders that greatly reduce patients’ quality of life^2^. At present, the commonly used treatments are microfracture technology, autologous or allogenic cartilage transplantation and autologous chondrocyte implantation (ACI)^3–5^. Although these methods can solve the problem of cartilage regeneration to a certain extent, most of the regenerated tissues formed by these methods are fibrous cartilaginous tissues; thus, it is difficult for these tissues to achieve the composition and mechanical properties akin to those of natural articular cartilage, and long-term efficacy is not guaranteed.

To solve this problem, numerous researchers have focused their attention on mesenchymal stem cells (MSCs). MSCs are pluripotent adult stem cells that exist in a variety of tissues, such as bone marrow (BMSCs)^6^, adipose tissue (ADSCs)^7^, synovial membrane (SDSCs)^8^, and umbilical cord Wharton’s jelly (WJMSCs)^9^. Owing to the capacity of MSCs for self-renewal, multidifferentiation, and immunoregulation, MSC-based therapy has great potential for cartilage regeneration^10^. According to a meta-analysis conducted by Hirotaka Iijima, the clinical application of MSCs is reasonably safe since no serious adverse events were reported, and MSCs delivered by intraarticular injection or arthroscopic implantation can significantly alleviate pain and improve knee function^11^.

However, basic and clinical studies of MSCs for the treatment of articular cartilage injury have also encountered some problems. Owing to the heterogeneity of MSCs, the influence of in vitro culture conditions and the inflammatory microenvironment in the joint cavity, some MSCs will exhibit an unstable cell morphology or undergo suboptimal chondrogenic differentiation and cartilage matrix formation or even rapid cell death. It has also been suggested that only some of the transplanted MSCs will eventually be effective, resulting in failure to obtain stable and homogeneous cartilage tissue or sustained therapeutic effects. In recent years, increasing attention has been focused on the heterogeneity of MSCs, which potentially reflects the diversity of MSCs in the range of embryonic origins, anatomical locations, biological properties, and functions^12^. Since heterogeneity poses a significant obstacle in the research and application of MSCs, it is of great significance to understand the heterogeneity of MSCs so that superior seed cells can be selected to bolster cartilage damage repair.

In this review, we first discuss the role of MSCs in cartilage regeneration, along with the heterogeneity of MSCs at three levels: the donor, tissue source, and cell subpopulation levels. Then, we summarize the potential of different MSCs for cartilage repair, including their capability for proliferation, chondrogenesis and immunoregulation, as well as their applications in cartilage regeneration.

## Role of MSCs in cartilage repair

**Fig. 1:**
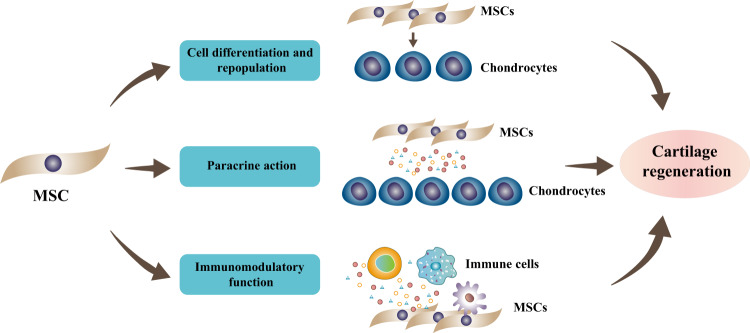
Schematic representation of the role of MSCs in cartilage regeneration. MSCs promote cartilage regeneration by diverse mechanisms. MSCs are able to proliferate and differentiate into chondrocytes to replace damaged cells. In addition, MSCs can also secrete cytokines to maintain chondrocyte phenotypes, promote their proliferation and ECM composition. More importantly, MSCs can exert immunomodulatory effects on diverse immune cells upon exposure to injured tissue or inflammatory factors.

Articular cartilage is hyaline cartilage, which is mainly composed of chondrocytes and extracellular matrix (ECM). Chondrocytes account for only ~5% of the total volume of articular cartilage and are responsible for maintaining the matrix. On the other hand, cartilage matrix accounts for the majority of the total volume of articular cartilage, and its main components are water, collagen type II (COL II) and proteoglycan^13^. Developing MSC-based strategies for cartilage repair would require an understanding of the mechanisms by which MSCs mediate tissue regeneration (Fig. 1).

Although it has been demonstrated that endogenous MSCs contribute to tissue repair, their quantity and potency are insufficient to completely regenerate damaged tissue^14^. On the other hand, it has been proposed that transplanted MSCs are able to migrate to sites of damage and then differentiate into target cells and replace damaged tissues^15^. Therefore, researchers have been working to find exogenous MSC populations with favorable potential for migration, proliferation, and chondrogenic differentiation for cartilage regeneration. For example, SDSCs have been reported to possess greater proliferative ability and chondrogenic potential than BMSCs and ADSCs, making them a focus of research in cartilage tissue engineering^16^.

Recently, an increasing number of researchers have indicated that in addition to differentiation and repopulation, MSCs can also achieve therapeutic effects through paracrine actions^17^. In terms of articular cartilage regeneration, MSCs can promote the proliferation and ECM deposition of chondrocytes by releasing trophic factors, such as transforming growth factor beta (TGF-β), insulin-like growth factor 1 (IGF-1), and thrombospondin 2 (TSP-2)^18^. In addition, when cocultured with MSCs, chondrocytes were able to maintain a stable mature phenotype with decreased expression of hypertrophic and fibrotic markers, which was partly due to the secretion of hepatocyte growth factor (HGF) by the MSCs^19^.

Furthermore, since damaged cartilage is exposed to a progressive inflammatory environment, the role of MSCs in modulating the immune response is also involved in the cartilage repair process. MSCs are believed to possess low immunogenicity and lead to a weak immune response due to their lack of expression of MHC class II and classic costimulatory molecules, such as CD80, CD86 and CD40^20^. Furthermore, after exposure to injured tissue or inflammatory cytokines, MSCs are capable of exerting immunomodulatory effects on diverse immune cells. It was reported that some cell surface molecules, such as programmed death-ligand 1 (PD-L1) and Fas ligand (FasL), mediated the immunomodulatory function of MSCs^21^. After binding to MSCs, the immune activities of T cells are inhibited^22^. In addition to cell contact, MSCs can also regulate immune cell function via the secretion of various cytokines. For example, IFN-γ upregulated indoleamine 2,3-dioxygenase (IDO) expression in MSCs via the JAK-STAT1 signaling pathway, which was involved in the inhibition of peripheral blood mononuclear cell (PBMC) proliferation and M2 macrophage polarization^23,24^. In the presence of interleukin-17A (IL-17A), MSCs expressed more PGE2 and could significantly increase the proportion of CD4^+^Foxp3^+^ Tregs and suppress T-cell proliferation^25^. In addition, MSCs are capable of secreting TGF-β and interleukin-6 (IL-6) to suppress the inflammatory responses of natural killer (NK) cells by inducing senescent-like NK cells^26^. Moreover, other molecules, such as nitric oxide (NO), inducible nitric oxide synthase (iNOS)^27^, human leukocyte antigen-G (HLA-G)^28^ and interleukin-10 (IL-10)^29^, have also been shown to mediate the immunosuppressive function of MSCs.

## Three levels of MSC heterogeneity

**Fig. 2:**
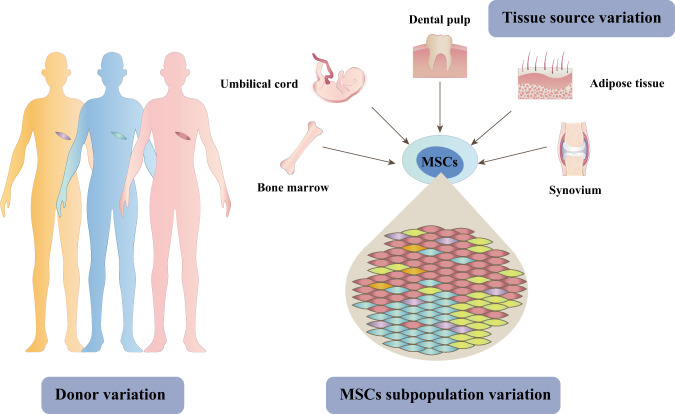
Overview of three levels of MSC heterogeneity. The heterogeneity of MSCs is inherent among donors, tissues and cell populations. The cytological function of MSCs from different donors is not identical. In addition, MSCs from different tissue sources are also quite different from each other in their cellular properties, such as proliferation, multiple differentiation and immunoregulation abilities. Moreover, MSCs from the same tissue are heterogeneous that not all of them share the same cellular functions.

MSCs are naturally plastic and exhibit differences in their phenotypes and functions. This heterogeneity is inherent among donors, tissues and cell populations (Fig. 2)^30–32^. Previous studies have reported that donor variation, including in terms of age^33^, sex^34^, and physiological status^35^, could result in functional differences in MSCs. Trivedi et al.^36^ compared the properties of BMSCs from different swine donors and found that their morphology, growth rate and trilineage differentiation potential were quite different from each other even though they shared comparable expression of some cell surface markers. In addition, Čamernik et al.^37^ observed that compared to those from healthy donors, bone-derived MSCs from osteoarthritis (OA) patients displayed a reduced trilineage differentiation potential, as well as significantly lower expression of CD73 and leptin receptors.

One of the well-recognized features of MSCs is their wide distribution in the body. Many studies have demonstrated that the functional characteristics of MSCs from different tissue sources are not identical. For example, BMSCs possess great osteogenic potential and tend to undergo chondrocyte hypertrophy^38^, while ADSCs are more likely to differentiate into adipocytes^39^. It was also reported that >1400 genes associated with tenogenic potential and chemotaxis were significantly different between human ADSCs (hADSCs) and human BMSCs (hBMSCs)^40^. WJMSCs, on the other hand, display significantly lower immunogenicity than MSCs from other tissues^41^.

MSCs from the same tissue are also heterogeneous, and not all of them share the same phenotypical markers and cellular functions. The Mesenchymal and Tissue Stem Cell Committee of the International Society for Cellular Therapy (ISCT) defined MSCs as expressing CD105, CD90, and CD73 and not expressing CD45, CD34, CD14 (CD11b), CD79 (CD19) or HLA-DR^42^. Later, many novel surface markers expressed on MSCs were found, which are more commonly used to identify a group of MSC subpopulations expressing different types of regulatory proteins that function in hematopoiesis, angiogenesis, and neural activities, as well as in immunity and defense-related processes^43^. These MSC subpopulations can be used to treat specific diseases according to their unique features to improve the therapeutic effect. For example, Du et al.^44^ verified that CD106^+^ placenta chorionic villi-derived MSCs (CVMSCs) had greater angiogenic paracrine activity than CD106^−^ CVMSCs, and the transplantation of CD106^+^ CVMSCs into the ischemic hind limb in a mouse model also resulted in more significant functional improvement. Likewise, some MSC subpopulations have proven to possess a stronger ability for proliferation, migration, chondrogenesis, or immunomodulation and have great value for the optimization of MSC-based cartilage regeneration strategies, which will be emphatically discussed later.

Among the MSC heterogeneity existing in donors, tissue sources, and cell subpopulations, it is hard to judge which one has the greatest impact on MSC properties. Few study has directly compared the three levels of MSC heterogeneity. Nevertheless, based on current researches, variability of MSC in different tissues sources seems to be more easily discovered and widely recognized as compared with that in different donors or cell subpopulations. In fact, MSC is known to originate from mesoderm and neural crest and its formation is spatially and temporally controlled by many factors during embryogenesis^45^. Besides, MSCs in specialized niches within different tissues are regulated by local microenvironments and hold some tissue-committed properties^46^. Even from topographically related tissues, MSCs also have different morphology, immunophenotype, and cellular functions. For example, Vasandan et al.^47^ compared the multipotency and immunomodulatoty potential of dental pulp derived MSCs (DPSCs) and periodontal ligament derived MSCs (PLSCs). They demonstrated that both DPSCs and PLSCs possessed favorable chondrogenesis, but only DPSCs showed adipogenic and osteogenic propensities; both DPSCs and PLSCs were hypo-immunogenic, but only DPSCs exhibited immunosuppressive effect on mitogen-induced lymphocyte proliferation.

## Differences among donors

Since the prevalence of OA increases with age, the correlation between the potential of MSCs for cartilage repair and donor age should be considered when selecting MSCs for cell-based therapy. In addition, whether MSCs from the bone marrow and synovial tissue of OA patients could serve as a potential cell type for autologous transplantation to treat cartilage damage remains unclear; thus, the properties of MSCs from OA patients and healthy people need to be compared directly.

### Age

Some studies have evaluated the cytological characteristics of MSCs from donors of different ages. Siegel and his colleagues isolated BMSCs from the bone marrow of 53 donors (28 males, 25 females; age: 13–80) and found that BMSCs derived from younger donors had a stronger ability for clone formation and immunoregulation^34^. Taguchi et al.^48^ reported similar results, demonstrating that ADSCs derived from young dogs could inhibit the proliferation of activated T lymphocytes more effectively than those derived from older dogs. In addition to immunomodulatory properties, the authors confirmed that the proliferative capacity also decreases with donor age, as indicated by the significantly longer in vitro doubling time of ADSCs derived from older dogs. Alt and coworkers^49^ found that the potential of ADSCs for proliferation and multidifferentiation was lower, while the expression of senescence-related genes, such as *CHEK1* and *ink4a*, was greater in aged donors than in young donors. Additionally, Kanawa et al.^50^ found that after chondrogenic induction, hBMSCs from young donors synthesized more GAG and expressed more *Sox9, COL II*, and *aggrecan* than hBMSCs from elderly donors, suggesting that the chondrogenic potential of hBMSCs decreased with donor age. In addition, they found that the osteogenic and adipogenic potential of hBMSCs was independent of the age of the donor. These studies suggest that the cellular functions of MSCs, such as colony formation, proliferation, chondrogenic differentiation and immunoregulation, all decrease with donor age.

Furthermore, Rauscher et al.^51^ reported that BMSCs from younger mice had a superior therapeutic effect on atherosclerosis than BMSCs from older mice. Similarly, Bruna et al.^52^ used BMSCs to promote cutaneous wound healing in mice and found that the reparative effect was poorer with BMSCs from older mice, indicating that MSCs from younger donors have better therapeutic effects. However, more animal experiments are needed to confirm whether BMSCs from young mice are more effective for cartilage repair.

### Osteoarthritis

As early as 1998, Oreffo et al.^53^ reported that OA does not affect the colony-forming ability of MSCs. Murphy et al.^54^ found that the proliferative, chondrogenic, and adipogenic capability of BMSCs from OA patients declined, while their osteogenic ability was similar to that of BMSCs from normal people. However, in this study, the average age of the OA patients (71 ± 7 years) was higher than that of the normal donors (43.5 ± 11.3 years), suggesting that there may be an age-related confounding factor. In another study, Fulber and his colleagues observed that the chondrogenic differentiation capacity of SDSCs from OA mice was weaker than that of SDSCs from healthy mice, as evidenced by the lower production of ECM after chondrogenic differentiation was induced^26^.

In contrast, some researchers have demonstrated that BMSCs from OA patients have a chondrogenic differentiation capacity similar to that of healthy donors. Dudics et al.^55^ isolated BMSCs from the bone marrow of OA patients (41–68 years old) and healthy volunteers (42–78 years old) and compared their chondrogenic differentiation capacity. After 14 days of in vitro chondrogenic induction, the synthesis of proteoglycan and expression of *COL II* not significantly differ between BMSCs from OA patients and BMSCs from healthy people, which is consistent with the earlier observations made by Scharstuhl and colleagues^56^.

In summary, MSCs from OA patients seem to possess a weaker proliferative ability, but it is controversial whether the chondrogenic ability of MSCs from OA donors is different from that of MSCs from healthy donors. Furthermore, since OA is a chronic inflammatory disease, it is necessary to further compare the immunoregulatory ability of MSCs from OA and healthy donors. However, few related studies have been reported. Thus, whether BMSCs or SDSCs from OA patients are appropriate for autologous transplantation to treat cartilage damage warrants further research.

In addition to age and OA, other characteristics of donors, such as sex, obesity and other physiological features, may also affect the functions of MSCs. It was reported that BMSCs from younger female donors possessed higher colony-forming potential^34^. Researchers also found that WJMSCs from obese donors exhibited decreased proliferation ability but greater immunosuppressive activity^57^. In addition, compared to SDSCs from healthy joints, SDSCs from joints with osteochondritis dissecans showed reduced proliferation and chondrogenic differentiation capacities^35^. All of these factors has certain value to the selection of appropriate donors for MSCs in cartilage regeneration. However, there are still some issues need to be addressed. Above all, these characteristics of donors may have different effects on MSCs from different tissue sources. Comprehensive studies comparing the effects of donor characteristics on different MSCs are needed. Besides, effects of donor characteristics on the ability of MSCs to repair cartilage in vivo is undetermined now and warrant further research.

## Tissue source-dependent variation in MSCs for cartilage repair

**Fig. 3:**
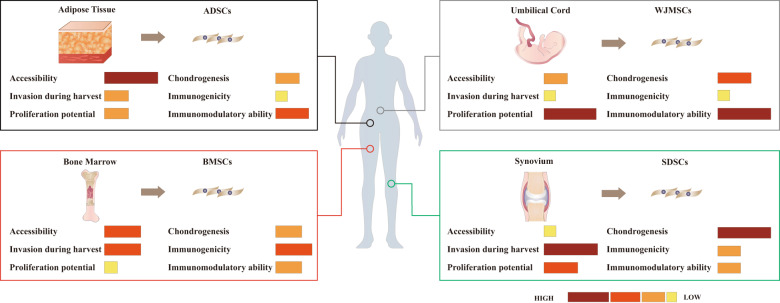
Comparison of cartilage potentials of MSCs derived from bone marrow, adipose tissue, synovium, and umbilical Wharton’s jelly. Although these MSCs may have similar morphology, their biological functions are quite different, which contribute to the heterogeneous cartilage repair potentials among them. Based on the date from previous researches, the accessibility, invasion during harvest, proliferation potential, chondrogenesis, immunogenicity, and immunomodulatory ability of MSCs form different tissue are compared with each other, which are visualized by color and length ranging from high (long brown band) to low (short yellow band).

Currently, BMSCs, ADSCs, SDSCs, and WJMSCs are four of the most widely used types of MSCs in cartilage tissue engineering, each with their respective advantages in applications for cartilage regeneration. However, there is heterogeneity in their potential for cartilage repair, including their accessibility, invasion during harvest, immunogenicity, and proliferative, chondrogenic, and immunomodulatory ability (Fig. 3).

### Bone marrow-derived mesenchymal stem cells

BMSCs are one of most studied seed cells in cartilage tissue engineering. It has been reported that BMSCs from the iliac crest and vertebral body are more prone to differentiate into chondrocytes than those from the femoral head^58^, indicating that the chondrogenic potential of BMSCs from different parts of the body varies. In addition, researchers found that hypoxia-cultured BMSCs underwent more chondrogenic differentiation than normoxia-cultured BMSCs^59^. Moreover, BMSCs have been shown to possess immunomodulatory functions. After being activated by inflammatory factors, BMSCs are able to secrete more IDO and promote M2 macrophage polarization, and these macrophages then tend to secrete more IL-10 and less IL-1β, resulting in better tissue survival in vivo^60,61^.

BMSCs have been commonly utilized to treat cartilage damage. For example, Jin et al.^62^ built a gradient construct composed of differently predifferentiated rabbit BMSCs (rBMSCs)-mesh to mimic the hierarchical complexity of osteochondral tissue, which effectively enhanced the reconstruction of osteochondral defects in rabbits. Sun et al.^63^ constructed a 3D-printed PCL scaffold containing GDF-5 PLGA microspheres and a rBMSC-loaded hydrogel. In the presence of GDF-5, these BMSCs further differentiated into articular chondrocytes, thus contributing to the superior effect of this composite on cartilage regeneration in rabbits. Some researchers seeded BMSCs on PGA/PLA scaffolds to induce chondrogenesis and to construct mature engineered cartilage in vitro. After biochemical and biomechanical evaluations, the superior composites were selected for animal experiments. This technique provides a new approach for treating cartilage lesions^64^.

The clinical application of BMSCs to repair cartilage damage has also been reported to be safe and effective. In 2013, Orozco et al.^65^ reported that the injection of autologous BMSCs was able to improve the knee function and cartilage quality of OA patients. Similarly, in a phase I-II clinical trial, 50 patients with chronic OA (median age = 52 years) received an autologous BMSC injection. After 1 year, magnetic resonance imaging (MRI) examinations revealed signs of cartilage regeneration in all patients, and their quality of life was improved^66^. Additionally, Chahal et al.^67^ found that autologous BMSCs were able to reduce synovial inflammation, as evidenced by decreased levels of proinflammatory monocytes/macrophages and IL-12, which could be predicted by a branch of anti-inflammatory markers of BMSCs, such as principal component 1 (PC-1) and TSG-6. In another study, it was determined that the long-term outcomes of OA patients treated by autologous BMSC transplantation were comparable to those of OA patients treated by ACI, while no increased tumor risk was found in the BMSC group^68^. Hashimoto et al.^69^ conducted a randomized controlled clinical trial to investigate the efficacy of autologous BMSC transplantation in combination with microfracture (MFX) (BMSC + MFX group, *n* = 7) compared with that of microfracture alone (control, *n* = 4). After 48 weeks of follow-up, they found that treatment with BMSCs + MFX was more efficacious in cartilage and subchondral bone healing and resulted in better outcomes than treatment with MFX alone. On the other hand, Vega et al.^70^ attempted to use an allogenic BMSC injection to treat chronic OA and found that its therapeutic effect was significantly better than that of hyaluronic acid, while no obvious adverse reaction was observed. Therefore, the treatment of cartilage defects with allogenic BMSCs is considered safe and effective. Nevertheless, new cartilage formed by injecting BMSCs into the knee joint is often structurally uneven. Biomaterials can effectively solve this problem by providing a framework for regenerated tissue. However, studies of biomaterial scaffolds combined with BMSCs for OA treatment are still in the animal stage, and more reports on their clinical applications are expected in the future.

### Adipose tissue-derived mesenchymal stem cells

Owing to their abundance, easy accessibility, and good capacity for in vitro proliferation and differentiation, ADSCs are widely utilized in cartilage regeneration^71,72^. Adipose tissue is acquired mainly from subcutaneous fat at present, while subpatellar fat obtained by arthroscopy could provide another source for ADSCs^73^. The chondrogenic differentiation potentials of MSCs from different tissues are not identical (Table 1). Many studies have indicated that the chondrogenic ability of ADSCs is inferior to that of BMSCs^74–76^, but ADSCs possess a greater proliferative capacity than BMSCs^39^. The cellular senescence of ADSCs occurs much later than that of BMSCs^77^. Moreover, ADSCs exhibit more potent immunoregulatory effects than BMSCs, as evidenced by greater IDO activity^78^.

**Table 1:** Phenotypic qualities and chondrogenic differentiation potential of MSCs from different tissues.

Cell type			Species	Induction method	Analysis method	Comparison	Ref
Type 1	Type 2	Type 3					
BMSCs	ADSCs		Human	Monolayer cultured and 3D pellet cultured in chondrogenic medium for 2 weeks	Cartilage related gene expression analysis, Safranin O staining, IHC staining for COL II	BMSCs = ADSCs in monolayer, BMSCs > ADSCs in 3D culture.	^74^
CD34^−^CD45^−^ CD49d^−^CD73^+^CD90^+^CD105^+^ CD166^+^ BMSCs	CD34^−^CD45^−^ CD49d^−^CD73^+^CD90^+^CD166^+^ADSCs		Human	3D pellet cultured in chondrogenic medium for 4 weeks	GAG content, COL I, COL II, Sox9 genes expression, Safranin O staining, IHC staining for COL II, IF analysis for Sox-9.	BMSCs > ADSCs	^75^
CD31^−^CD34^−^ CD45^−^ HLA-DR^−^ CD29^+^CD44^+^CD90^+^CD105^+^HLA-ABC^+^ BMSCs	CD31^−^CD34^−^ CD45^−^ HLA-DR^−^ CD29^+^CD44^+^CD90^+^CD105^+^HLA-ABC^+^ ADSCs		Human	3D pellet cultured in chondrogenic medium for 3 weeks	IHC staining for COL II	BMSCs > ADSCs	^76^
CD11b^−^CD45^−^CD44^+^CD90^+^ SDSCs	CD11b^−^CD45^−^CD44^+^ BMSCs	CD11b^−^CD45^−^CD44^+^ ADSCs	Canine	3D pellet cultured in chondrogenic medium for 3 weeks	sGAG content	SDSCs = BMSCs > ADSCs	^16^
SDSCs	BMSCs	ADSCs	Porcine	Cells were seeded on hydrogels and cultured in chondrogenic medium for 7 weeks	sGAG and collagen content	SDSCs > ADSCs > BMSCs	^38^
CD11b^−^CD45^−^CD90^+^ SDSCs	CD11b^−^CD45^−^CD90^+^ BMSCs	CD11b^−^CD45^−^CD90^+^ ADSCs	Rat	3D pellet cultured in chondrogenic medium for 3 weeks	Pellets weight, toluidine blue staining, COL II gene expression, chondroitin sulfate and hyaluronan content	SDSCs > BMSCs > ADSCs	^96^
CD34^−^CD45^−^ HLA-DR^−^ CD44^+^CD73^+^CD90^+^CD105^+^WJMSCs	CD34^−^CD45^−^ HLA-DR^−^ CD44^+^CD73^+^CD90^+^ BMSCs		Human	Cells were seeded on hydrogels and cultured in chondrogenic medium without added growth factor for 4 weeks	Cartilage-specific transcript expression analysis, Alcian blue staining, IF and IHC staining for COL II	WJMSCs > BMSCs	^109^
WJMSCs	BMSCs		Human	Cells were seeded on PGA scaffolds and cultured in chondrogenic medium without added growth factor for 6 weeks	sGAG content, IHC staining for COL I and COL II	WJMSCs > BMSCs	^110^

*BMSCs* bone marrow-derived mesenchymal stem cells, *ADSCs* adipose tissue-derived mesenchymal stem cells, *SDSCs* synovial membrane-derived mesenchymal stem cells, *WJMSCs* Wharton’s jelly-derived mesenchymal stem cells, *3D* three dimensional, *IHC* Immunohistochemical, *IF* immunofluorescence, *COL II* type II collagen, *COL I* type I collagen, *=* similar to, *>* greater than.

The effectiveness of ADSCs for cartilage regeneration in animal models has been verified. It was demonstrated that both ADSCs and three dimensionally cultured ADSC spheroids were able to promote the regeneration of injured cartilage. After in situ implantation, mouse ADSCs (mADSCs) could significantly reduce the proliferation and migration of inflammatory cells, as well as the production of inflammatory cytokines, in a rheumatoid arthritis mouse model. However, ADSC spheroids were able to produce more TGF-β1 and showed a lower rate of apoptosis, demonstrating their potential as a method for scaffold-free cartilage tissue engineering^79,80^. Dubey reported that mADSCs were capable of inhibiting the inflammatory cascade mediated by glycosylation in a diabetic OA mouse model, as shown by the decreased expression of CML, RAGE, MDA, NF-κB, MMP-1, and MMP-13 in the articular cavity^81^. When rabbit ADSCs (rADSCs) or their secretome were injected into the knees of rabbits with cartilage lesions, better cartilage regeneration was achieved in the ADSC group than in the secretome group^82^, indicating that ADSCs may be more effective in the repair of cartilage damage than their secretome. In addition, many types of biomaterial scaffolds based on chitosan, fibrin, atelocollagen, and decellularized cartilage have been used as carriers to provide a 3D culture environment for ADSCs, which could support the survival, proliferation and differentiation of ADSCs^83,84^. For example, Boyer et al.^85^ seeded hADSCs on an injectable hydrogel composed of silanized hydroxypropyl cellulose to repair osteochondral defects in dogs and demonstrated satisfactory effects after 4 months.

Currently, several clinical trials of ADSCs for treating cartilage damage have been reported. Lee et al.^86^ revealed that the intraarticular injection of autologous ADSCs resulted in satisfactory improvement in pain and symptoms in patients with OA at the 6-month follow-up. In addition, Kim et al.^87^ attempted injecting ADSCs in OA patients during the process of high tibial osteotomy. After almost 3 years, they found that the clinical outcomes of patients who received an ADSC injection were significantly better than those of patients treated with high tibial osteotomy alone. Pers et al.^88,89^ confirmed that ADSCs were capable of encouraging the transition of immune cells toward an anti-inflammatory phenotype in the knee cavity. They found that low-dose (2 × 10^6^ cells) ADSC transplantation improved the pain experienced by the patients and the knee function of patients with severe OA more than high-dose (5 × 10^7^) ADSC transplantation. However, in another randomized controlled clinical trial, researchers found that the reparative effect was better in the high-dose group (1 × 10^8^ cells) than in the low-dose group (1 × 10^7^ cells) and medium-dose group (5 × 10^7^ cells)^90^. A similar result was obtained by Song and his coworkers^91^. The optimal amount of ADSCs injected into the knee to achieve the best reparative effect still needs further exploration. Although the number of patients was limited, these studies reinforced the safety and efficacy of autologous ADSC therapy for cartilage damage. More large-scale and long-term clinical trials will help further promote the clinical application of ADSCs for cartilage regeneration.

### Synovial membrane-derived mesenchymal stem cells

SDSCs were first isolated from the human synovial membrane in 2001^92^. Researchers found that synovial fluid and arthroscopic trocar shaver blade filtrate also contained SDSCs. Arthrocentesis is less invasive and more convenient than synovial biopsy, but the proliferative capacity of SDSCs isolated from synovial fluid is slightly lower than that of SDSCs isolated from the synovial membrane or arthroscopic trocar shaver blade filtrate. Nevertheless, since synovial tissue displays a high regenerative ability, the source of SDSCs is still relatively abundant^93,94^.

Various studies have proven that SDSCs possess a stronger capacity for proliferation and chondrogenic differentiation than BMSCs and ADSCs^16,38,95,96^, while their osteogenic capability has been shown to be weaker than that of BMSCs. In addition, the expression of MSC markers (CD105, CD90, CD73, MHC-I) was similar between SDSCs and BMSCs, but MHC-II expression was much lower in SDSCs^97^, indicating that SDSCs also possessed an advantage in terms of immunogenicity. Djouad reported that the immunomodulatory ability of SDSCs was comparable to that of BMSCs. SDSCs were able to significantly inhibit T-cell proliferation in a mixed lymphocyte reaction and produced an amount of IDO similar to that produced by BMSCs after being stimulated by inflammatory factors^98^. Moreover, in view of the specific anatomical location, the effect of SDSCs on chondrocytes has been emphasized. Kim et al.^99^ cultured SDSCs with chondrocytes and observed significantly higher *COL II* and *Sox9* expression and lower *COL I* and *COL X* expression in the coculture group than in the chondrocyte monoculture group, suggesting that the coculture of SDSCs and chondrocytes could promote the deposition of ECM and inhibit the hypertrophy and osteogenic differentiation of chondrocytes. In addition, Pei et al.^100^ demonstrated that the ECM deposited by SDSCs during in vitro culture could promote chondrocyte proliferation and the cells could maintain their potential for redifferentiation.

Above all, SDSCs have been proposed as desired seed cells to induce neotissue with an abundant cartilage-specific content in the process of cartilage regeneration. Koga et al.^101^ transplanted rabbit SDSCs (rSDSCs) into the articular cartilage defect area of rabbits and covered them with periosteum. Histological staining and transmission electron microscopy revealed that the transplanted rSDSCs differentiated into chondrocyte-like cells and continuously synthesized ECM. Lee et al.^102^ encapsulated rSDSCs in a type I collagen/hyaluronic acid/fibrinogen composite and transplanted them into a rabbit knee with cartilage damage. The cartilage defect was found to be covered by hyaline cartilage with a high content of GAG and COL II after 24 weeks. Clinically, Sekiya et al.^103^ attempted the transplantation of hSDSCs in 10 patients with articular cartilage lesions. The reparative effects were satisfactory after 3 years or more, as indicated by MRI quantification, histological analysis and clinical evaluations. In a 2-year randomized study, 14 patients with full-thickness chondral lesions underwent autologous hSDSC implantation (AMI) or ACI. It was revealed that the functional outcome and quality of life were significantly better in the AMI group than in the ACI group^104^.

These studies show that SDSCs have a dramatic effect on the repair of cartilage damage and are promising in cartilage tissue engineering. However, studies on SDSCs are not as thorough as those on BMSCs and ADSCs, and more advanced biomaterials are needed to enhance the regenerative ability of SDSCs. Moreover, larger-scale clinical trials are needed to verify the superior potential of SDSCs for cartilage regeneration.

### Wharton’s jelly-derived mesenchymal stem cells

In 1991, McElreavey et al.^105^ first isolated WJMSCs from a normal human umbilical cord (38–44 weeks). Since the umbilical cord is usually discarded at birth, the collection of Wharton’s jelly is noninvasive and ethically uncontroversial, which provides an adequate source for WJMSCs. Furthermore, it can also be cryopreserved for autologous applications in the treatment of congenital or adult diseases.

In the past decades, the biological characteristics and functions of WJMSCs have been investigated in many studies. A comparative analysis of hWJMSCs and human embryonic stem cells (hESCs) using a DNA microarray showed that the transcriptomic profiles of hWJMSCs and hESCs were quite different. Some stem cell markers of hESCs, such as *POUF1*, *NANOG*, *SOX2* and *LIN28*, were expressed at low levels in hWJMSCs, which partly explained why the transplanted hWJMSCs did not induce teratoma formation^106^. Meanwhile, genes related to proliferation, adhesion and immunoregulation were highly expressed in hWJMSCs^107^. As an intermediate form between embryonic and adult stem cells, WJMSCs have been reported to have a greater capacity for proliferation and chondrogenic differentiation than BMSCs^108–110^. More importantly, WJMSCs are characterized by low immunogenicity and an excellent immunoregulatory ability, and these traits are maintained even after their differentiation into mature phenotypes^41^. It has also been reported that WJMSCs do not express classic costimulatory molecules, such as CD80, CD40 and CD86, but express immunoregulatory molecules, such as HLA-G, IDO, PGE2, HGF and IL-10, and could significantly suppress the proliferation of concanavalin A-stimulated lymphocytes. Furthermore, no obvious immune rejection was observed when hWJMSCs were subcutaneously transplanted into rats^111–113^. Thus, the immune properties of WJMSCs make them a great option for repairing damaged cartilage.

In small-animal OA models, the injection of WJMSCs into the damaged cartilage reduced cartilage fibrillation as well as MMP-1, MMP-3 and MMP-13 expression^114^, and TUNEL activity while increasing COL II, IGF-1 and TGF-β1 expression^115^, thus producing satisfactory reparative effects. Additionally, Zhang et al.^116^ attempted the coculture of hWJMSCs and primary cartilage cells in an ECM scaffold to repair damaged cartilage in goats. After 9 months, they found that hWJMSCs were one part of the neotissue, which was similar to native cartilage and positive for HLA-ABC on immunofluorescence staining, indicating that the transplanted hWJMSCs could evade surveillance of the caprine immune system and participate in cartilage regeneration. Clinical trials of WJMSCs for the treatment of acute myocardial injury^117^ and type 1 diabetes^118^ have been carried out. For cartilage injury repair, Sadlik et al.^119^ transplanted a hWJMSC-collagen I/III composite scaffold into the site of cartilage injury in the knee joint under dry arthroscopy, and MRI examination after 9 months showed that the regenerative tissue was well-integrated with the surrounding cartilage and subchondral bone. It is undeniable that WJMSCs are one of the most promising seed cells for cartilage tissue engineering, although additional high-quality and large-scale randomized controlled trials are needed.

In summary, although BMSCs and ADSCs are widely used in cartilage tissue engineering, SDSCs and WJMSCs have shown better cellular functions. A comprehensive analysis of the potential for cartilage regeneration, including the capacity for proliferation, chondrogenic differentiation and immunomodulation, indicates that WJMSCs are probably the most effective cell type in cartilage regeneration. However, their extensive application is still limited by inadequate resources. In turn, with the view of clinical application, ADSCs is still most promising for cartilage regeneration due to its abundant source, little invasion during harvest, and verified safety and effectiveness. In addition, MSCs derived from other tissues, such as the periosteum^120^, amniotic membrane^121^, peripheral blood^122^, and dermis^123^, have also been identified. More research on these MSCs is needed to evaluate whether they are suitable for the repair of damaged articular cartilage.

## Potential of MSC subpopulations for cartilage repair

**Fig. 4:**
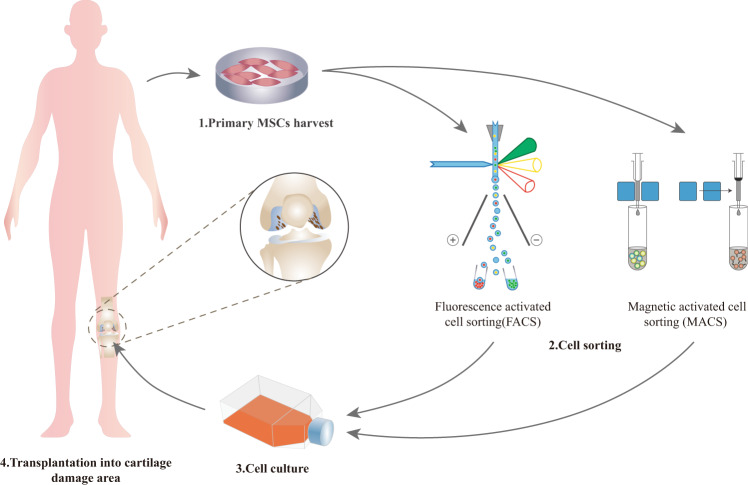
The schema diagram of MSCs subpopulation-based therapeutic strategy for cartilage damage. (1) Primary MSCs are harvested from various tissue in the body. (2) After expanding for 2 or 3 passages, MSCs subpopulations are isolated using surface markers such as CD271, CD146, CD49f, CD105 and Stro-1 via fluorescence activated cell sorting (FACS) or magnetic activated cell sorting (MACS). (3) Isolated MSCs subpopulations are cultured for 24–72 h to remove the dead cells caused by separating process. (4) MSCs subpopulations with superior cartilage repair potentials are transplanted into cartilage damage area so as to improve the treatment outcome.

The selection and utilization of functional MSC subpopulations for the treatment of cartilage damage has gradually become a new area of research focus (Fig. 4). Among various MSC subpopulations, CD271^+^, CD49f^+^, CD146^+^, CD105^+^, and Stro-1^+^ MSCs show great prospects for further improvement of cartilage repair. Table 2 lists the potential of different MSC subpopulations for cartilage regeneration.

**Table 2:** Cartilage regeneration potential of different MSC subpopulations.

Cell type	Control	Species	In vitro or in vivo	Cartilage regeneration potential	References
CD271^+^ BMSCs	CD271^−^ BMSCs	Human	In vitro	Showed greater clonogenic and proliferation capacity	^129^
	PA BMSCs	Human	In vitro and in vivo	Possessed greater chondrogenic potential both in vitro and in vivo and obtained higher histological scores when used for cartilage damage repair in rats	^130^
	PA BMSCs	Human	In vitro	Showed greater immunosuppressive ability	^132,133^
CD271^+^ ADSCs	PA ADSCs	Human	in vitro	Displayed greater CFU-F activity, proliferation and trilineage differentiation potential	^127^
	PA ADSCs	Human	In vitro and in vivo	Demonstrated less angiogenic potential in vitro and in vivo and performed better in early stage tissue repair of osteochondral lesions in rats	^131^
CD271^+^ SDSCs	CD271^−^ SDSCs	Human	in vitro	Promoted greater filling, achieved better integration and obtained higher histologic scores when used for in vitro repair of human cartilage defects	^126^
CD49f^+^ BMSCs	CD49f^−^ BMSCs	Human	In vitro	Demonstrated better proliferation and adhesion capacity	^144^
CD49f^+^ UCBSCs	CD49f^−^ UCBSCs	Human	In vitro	Possessed stronger proliferation ability	^145^
CD146^+^ PLSCs	CD146^−^ PLSCs	Human	In vitro	Showed greater proliferation ability	^151^
CD146^+^ UCMSCs	CD146^−^UCMSs	Human	In vitro	Had longer telomere length	^154^
	CD146^−^ UCMSs	Human	In vitro and in vivo	Suppressed Th17 cell activation and attenuated the progression of collagen induced arthritis in mice	^158^
CD146^+^ BMSCs	CD146^−^ BMSCs	Human	In vitro	Produced more sGAG after chondrogenic induction	^155^
CD146^+^ ADSCs	PA ADSCs	Human	In vivo	Reduced early inflammatory response in the knee cavity in rats and repaired cartilage damage in rabbit more effectively	^159^
CD105^+^ SDSCs	CD105^−^ SDSCs	Human	In vitro	Possessed greater chondrogenesis	^164,165^
	PA SDSCs	Rat	In vitro	Expressed more *COL II* and *Sox9* after 3D chondrogenic induction culture	^166^
CD105^−^ ADSCs	CD105^+^ADSCs	Mouse	In vitro	Displayed greater immunoregulation capacity	^167^
Stro-1^+^ BMSCs	PA BMSCs	Human	In vitro	Possessed stronger clone forming, proliferation, multiple differentiation, and paracrine activity	^152^

*BMSCs* bone marrow-derived mesenchymal stem cells, *PA* plastic adherence, *ADSCs* adipose tissue-derived mesenchymal stem cells, *CFU-F* fibroblast colony-forming unit, *SDSCs* synovial membrane-derived mesenchymal stem cells, *COL II* type II collagen, *UCBSCs* umbilical cord blood-derived mesenchymal stem cells, *PLSCs* periodontal ligament stem cells, *UCMSCs* umbilical cord-derived mesenchymal stem cells.

### CD271

CD271, also known as low-affinity nerve growth factor receptor (LNGFR), was initially found to be expressed in neurons, Schwann cells, melanocytes and follicular dendritic cells. Later, some scholars found that CD271 was detected in cells derived from mesenchymal, epithelial and hematopoietic tissues^124^. In the case of bone marrow or adipose tissue, CD271 could be considered a suitable marker for the isolation of MSCs. A high number of CD271^+^ cells were found in BMSCs as well as in ADSCs, while fetal MSCs, such as cord blood MSCs (CBMSCs) and WJMSCs, showed very low or no CD271 expression^125^. Interestingly, in the synovial tissue of OA patients, cells expressing CD271 were mainly located in the subintimal band, and the proportion was higher than that observed in healthy people^126^.

Further, some researchers found that compared with unsorted MSCs, CD271^+^ ADSCs and CD271^+^ BMSCs showed greater clonogenic, proliferative and differentiative potential^127–130^. Meanwhile, CD271^+^ ADSCs were less angiogenic and had less influence on endothelial cell migration or endothelial tubule formation both in vitro and in vivo^131^. In addition, compared with plastic-adherent (PA) BMSCs, CD271^+^ BMSCs have greater immunoregulatory potential and can significantly inhibit the mitogenic-induced proliferation of PBMCs due to increased PEG2 production^132,133^. For SDSCs, the CD271^+^ subpopulation displayed greater chondrogenic potential than the CD106^+^ or CD73^+^ subpopulation, as evidenced by increased expression of *COL II* and *aggrecan* after chondrogenic differentiation^134^.

Based on these results, it is proposed that CD271^+^ MSCs have advantages in terms of proliferation, differentiation and immunoregulation. At present, CD271^+^ MSCs have been employed to repair articular cartilage defects and have yielded better results than CD271^−^ or PA MSCs. Mifune et al.^130^ utilized the transplantation of CD271^+^ or PA hBMSCs to repair cartilage defects in rats and found glossy white and well-integrated repaired tissues in both the CD271^+^ hBMSC and PA hBMSC groups. However, higher histological scores and chondrocyte marker expression levels, as well as fewer apoptotic chondrocytes, were observed in the CD271^+^ group. When CD271^+^ and CD271^−^ hSDSCs were implanted into human cartilage defects and placed in chondrogenic differentiation induction medium for in vitro repair, 8 weeks later, more tissues positive for safranin O staining and better integration of the regenerative tissue and normal tissue were found in the CD271^+^ SDSC group^126^.

However, the signaling pathways and mechanisms through which CD271 affects the cytological properties of MSCs remain unclear. In addition, the use of CD271^+^ MSCs as better seed cells in cartilage tissue engineering still lacks data for in vivo large-animal experiments.

### CD49f

CD49f, also known as integrin α6, is involved in cell–cell and cell–matrix interactions^135^. CD49f is widely expressed on the surface of various stem cells, including hematopoietic stem cells^136^, neural stem cells^137^, breast stem cells^138^, ESCs^139^, tumor stem cells^140^, and MSCs^141^. CD49f^+^ stem cells have been proven to have a superb ability for proliferation and multidifferentiation^142^.

In terms of MSCs, some researchers found that the expression of CD49f in DPSCs was higher than that in PLSCs, and the DPSCs showed a greater capacity for proliferation and differentiation than the PLSCs^143^. The proportion of CD49f^+^ cells in fetal hBMSCs is much higher than that in adult hBMSCs and gradually decreases with increased passaging in vitro. In addition, the expression of CD49f is sensitive to environmental changes and is decreased in the inflammatory environment. Compared with CD49f^−^ BMSCs, CD49f^+^ BMSCs possess greater potential for proliferation and adipogenic and osteogenic differentiation^144^, which are closely related to activation of the PI3K/AKT/p53 pathway^145^. Additionally, it has been reported that CD49f^+^ bone, cartilage, and stromal progenitor cells (BCSPs) have greater potential for clone formation and multidifferentiation and that the proportion of CD49f^+^ BCSPs is significantly increased after fracture^146^, indicating that CD49f^+^ BCSPs play an important role in fracture healing. On the other hand, the proportion of CD49f^+^ MSCs in bone marrow in patients with OA is higher than that in patients with fracture^147^, but whether they are involved in the progression of OA has not yet been investigated.

These results show that CD49f^+^ MSCs possess stronger “stemness” than CD49f^−^ MSCs. However, few studies on the chondrogenic differentiation potential of CD49f^+^ MSCs and their potential as seed cells for cartilage tissue engineering have been reported. In addition, given that the expression of CD49f gradually decreases with increased passaging in vitro, CD49f^+^ MSCs are considered to have low immunogenicity, but there is still a lack of relevant research.

### CD146

CD146, also known as melanoma cell adhesion molecule (MCAM), is a transmembrane glycoprotein expressed on pericytes and endothelial, smooth muscle, stromal, and perivascular MSCs, which are involved in tissue maintenance and regeneration^148^. In human periapical cyst MSCs (hPCyMSCs), the expression of CD146 declines during passaging at both the mRNA and protein levels, and the CD146^low^ subpopulation has greater potential for proliferation and osteogenic differentiation^149^. It was also reported that BMSCs with low CD146 expression proliferated faster than BMSCs with high CD146 expression; however, there was no significant difference in differentiation potential between them^150^. Controversially, CD146^+^ PLSCs were reported to have a greater capacity for proliferation and multilineage differentiation^151,152^. MSCs from the umbilical cord with self-renewal and multidifferentiation potential can also be isolated and sorted by the CD146 marker^153,154^, and the telomeres of CD146^+^ hUCMSCs were longer than those of CD146^−^ hUCMSCs^154^. In addition, after induction in vitro, CD146^+^ hBMSCs produced more sGAG^155^. However, CD146 was reported to be involved in an outside-in signaling pathway, including the protein tyrosine kinases FYN, FAK and paxillin^156^; notably, FAK has been proven to be a negative regulator of chondrogenesis^157^.

The application of CD146^+^ subpopulations for cartilage regeneration in animal models has also been conducted. Wu et al.^158^ demonstrated that CD146^+^ hUCMSCs were more potent for cartilage regeneration than CD146^−^ hUCMSCs due to their greater immunoregulatory ability. When injected into the knee cavity of mice with arthritis, CD146^+^ hUCMSCs inhibited the activation of Th17 cells and protected cartilage more effectively than CD146^−^ MSCs, thus achieving a better reparative effect. Similarly, CD146^+^ hADSCs injected into the knee cavity of rats with osteochondral defects were more effective in reducing the early inflammatory response than PA hADSCs. In addition, when used to repair rabbit knee cartilage defects, CD146^+^ ADSCs in combination with articular cartilage ECM scaffolds could promote better regeneration in the long term^159^. Moreover, the use of autologous CD146^+^ perivascular cells in the repair of cartilage defects in the sheep knee joint has also achieved satisfactory results^160^.

In short, the CD146^+^ MSC subset has a superior immunoregulatory ability and can effectively reduce the early inflammation caused by cartilage injury, but its ability for proliferation and potential for chondrogenic differentiation are still controversial and need to be further studied. Nevertheless, some studies have used CD146^+^ MSC subsets as seed cells for cartilage damage repair, and the reparative effects were better than those of CD146^−^ MSCs or PA MSCs.

### CD105

CD105, also known as endoglin or TGF-β receptor III, is widely expressed on the surface of various MSCs^161,162^. Cells in the CD105^+^ subset isolated from bone marrow cells were CD73^+^, CD90^+^ and CD45^−^ and had the potential for self-renewal, multidifferentiation and immunoregulation^163^. It has been reported that CD105 on MSCs is involved in the TGF-β/Smad2 signaling pathway and in the regulation of chondrogenic differentiation. The chondrogenic potential of CD105^+^ SDSCs is stronger than that of CD105^−^ SDSCs^164,165^. Qi et al.^166^ seeded CD105^+^ SDSCs and PA SDSCs onto chitosan-alginate scaffolds for 2 weeks of chondrogenic induction in 3D culture and found that CD105^+^ SDSCs could express more *COL II* and *Sox9*. In addition, Anderson et al.^167^ found that CD105^+^ mADSCs shared similar growth potential with CD105^−^ mADSCs but that CD105^−^ mADSCs underwent more adipogenic and osteogenic differentiation. In terms of immunoregulatory capacity, CD105^−^ mADSCs seem to perform better than CD105^+^ mADSCs, which could express more iNOS and IL-6 after stimulation by TNF-α and IFN-γ and could inhibit the proliferation of splenocytes more effectively. Although the CD105^+^ MSC subset has a better chondrogenic capacity and greater potential for cartilage damage repair than other MSC subsets, few studies evaluating CD105^+^ MSCs for cartilage repair have been reported, and further research is urgently needed.

### Stro-1

The expression of Stro-1 is a characteristic of immature precursor cells^168^ and has been used to identify and isolate MSCs in a variety of tissues^169^. Koyama et al.^170^ found that SDSCs in human synovial fluid expressed Stro-1 and were capable of expanding in vitro and differentiating into several cell types, including osteoblasts, chondrocytes, adipocytes, and neurons. Xu et al.^152^ isolated tendon progenitor cells from human tendon cells by using the cell surface markers Stro-1 and CD146, which have a high colony-forming efficiency and multilineage differentiation potential. These tendon progenitor cells have chondrogenic and osteogenic differentiation potential equal to that of BMSCs, but their ability to differentiate into adipose tissue is weaker than that of BMSCs. It was reported that with increased passaging in vitro, the expression of Stro-1 decreased gradually^171^. In addition, CD146-Stro-1- SDSCs can be induced into SDSC-iPSCs by reprogramming techniques with *Sox2*, *Oct-3/4*, *klf4* and *c-Myc*, which express CD146 and Stro-1 again and exhibit improved proliferative activity and differentiation potential^95^. Additionally, Psaltis et al.^172^ reported that Stro-1^+^ BMSCs possessed stronger clone formation and proliferation abilities, multidifferentiation potential and paracrine function than PA BMSCs. Although Stro-1 is a surface marker of various MSCs, heterogeneity still exists in subpopulations sorted by Stro-1 alone. Thus, Stro-1 may need to be used in combination with other specific MSC surface markers to generate more homogeneous populations^173^.

### Single-cell RNA sequencing

Currently, the identification of MSC subpopulations with a strong potential for cartilage repair mainly relies on known cell markers related to proliferation, differentiation and inflammatory regulation. This method is not targeted and cannot be used to screen the functional subsets of MSCs. Single-cell transcriptome sequencing (scRNA-seq) has become a popular technique in recent years that can be used to perform MSC clustering based on specific markers and comparative analysis of different MSC subsets. It can not only accurately reflect the differences among MSC subsets but also be used to deeply explore the relationship between their genotypes and phenotypes, providing a new method for the screening of functional MSC subgroups^174^. For example, aiming at the heterogeneity existing in traditionally isolated tendon stem/progenitor cells (TSPCs) and the lack of a subpopulation of TSPCs that can contribute to tenogenesis, Yin et al.^175^ successfully identified a type of nestin^+^ TSPC with a strong ability for proliferation and tenogenic differentiation by utilizing single-cell transcriptome sequencing. The authors confirmed that nestin played a key role in the phenotypical maintenance and differentiation of TSPCs in vivo. Similarly, single-cell transcriptome sequencing also has great value for application in screening MSC subsets with superior proliferation, chondrogenic differentiation or inflammatory regulation.

Some researchers have performed scRNA-seq on mouse bone marrow stromal (BM) cells. Freeman and his coworkers revealed that all BMSCs exhibited high expression of multipotency-associated genes. However, genes associated with BMSC multilineage differentiation, including chondrogenic differentiation, and immunomodulation, showed variable levels among individual cells, which were independent of their proliferation status^176^. Specifically, Tikhonova et al.^177^ found that the Col2.3^+^Fbn1^high^gf1^high^ subpopulation expressed both osteogenesis and chondrocyte-specific genes, which may represent cells undergoing osteogenic transdifferentiation. In addition, Baryawno et al.^178^ distinguished four MSC subsets in mouse BM cells by the expression of *Cxcl12*, *Lepr*, *Grem1*, and other genes. One subset expressed significantly higher levels of functional osteolineage cell-specification genes *Sp7* and *Alpl*. In recent years, scRNA-seq has been conducted on cells from human beings^179^. Wang et al.^180^ utilized scRNA-seq to uncover in vivo heterogeneity of human BMSCs. They identified LEPR^hi^CD45^low^ BMSCs within freshly isolated CD271^+^ BM mononuclear cells. For further analyses, the BMSCs were divided into different subtypes and one of them were defined as chondrocyte precursor with expression of CD56 and WIF1. Besides, they suggested that there are considerable differences in the gene expression and differentiation trajectory betwwen mBMSCs derived chondrocyte precursor and hBMSCs derived chondrocyte precursor^180^. These results support and extend the concept of MSC heterogeneity at the individual cell level. Furthermore, on the basis of gene expression, scRNA-seq can be combined with the FACS technique to sort MSC subpopulations for cartilage regeneration.

Over all, applying MSCs subpopulations has shown great prospect in improving its therapeutic effects for cartilage regeneration. Among various MSC subpopulations, CD271^+^ MSC subpopulation has been the most studied one, which exhibited superior clonogenic, proliferative, and differentiative potential and desirable cartilage regeneration ability in vivo. On the other hand, CD146^+^ MSC subpopulation was reported to possess great immunomodulatory function and also performed well in cartilage regeneration in animal models. At this point, it is still hard to determine whether either is better than the other, but they may be used according to the status of cartilage injury. Besides, there would be much more functional MSC subpopulations in cartilage tissue engineering with the popularization and development of scRNA-seq. However, one remaining roadblock is that the cell isolation, no matter through fluorescent activated cell sorting (FACS) or magnetic activated cell sorting (MACS), could result in a degree of loss of cell count and viability. Thus, the selected MSC subpopulation should make up a proper proportion of the total MSCs and the sorting methods need to be improved.

## Perspectives

Over recent years, the concept of “precision medicine” has gained increasing attention due to its extensive scientific and political perspectives^181^. An important aspect of precision medicine is to provide the right drug to the right patient at the right time and dose according to the unique characteristics of each patient to maximize the efficacy of the drug and minimize adverse reactions^182^. In the context of MSC-based cartilage regenerative therapy, the heterogeneity of MSCs makes it difficult to obtain stable and effective therapeutic outcomes. Obtaining a complete picture of the potential of various MSCs for cartilage repair will facilitate the selection of appropriate seed cells, which will be conducive to improving the precision of MSC-based therapy for cartilage damage.

Currently, more and more researchers turn their attention to the exploration and application of functional MSC subpopulation. In this review, we systematically reviewed five widely studied, novel MSC markers and their effects on the cytological functions and potential of MSCs for cartilage repair. In addition, there are some less studied molecules that may have similar functionality. For example, GDF-5, also known as BMP-14, is a member of the TGF-β superfamily. GDF-5^+^ cells play an important role in joint formation and cartilage development. It has been reported that GDF-5^+^ SDSCs still exist in adult mice and proliferate significantly when cartilage is damaged^183^, but whether GDF-5 can be used as a marker of MSC subpopulations with greater chondrogenic potential needs further exploration.

The applications of some MSC subpopulations for cartilage repair have been proven to have better therapeutic outcomes and show great prospects in cartilage regeneration. However, there are still some drawbacks that limit their clinical applications. First, most of the relevant in vivo studies were performed in small animals, and more large-animal experiments are needed to thoroughly evaluate the preclinical safety and efficacy of these approaches. In addition, the expression of these novel markers seems to be susceptible to an inflammatory environment and tends to gradually decrease with passaging in vitro^144^. Developing new biological scaffolds that can help maintain the cellular phenotypes of MSCs may contribute to solving this problem. In addition, a specific marker must be expressed on MSCs to a certain extent to facilitate isolation of sufficient quantities of cells. Finally, the upstream and downstream gene expression profiles and signaling pathways of some specific markers are still unclear, thus hindering advancements regarding the therapeutic concept and method of using MSC subsets to repair cartilage damage. CD105 activates the TGF-β/Smad2 signaling pathway to enhance the chondrogenic capacity of SDSCs, and CD49f could regulate the differentiation potential and maintain the pluripotency of MSCs through the PI3K/AKT/P53 signaling pathway. More molecules and signaling pathways warrant further study to develop a sound theoretical foundation for using MSC subsets to repair damaged cartilage.

Since heterogeneity pose a significant obstacle to the application of MSCs, it is essential to develop strategies to reduce this heterogeneity. As illustrated herein, the heterogeneity of MSCs arises from variations in donors, tissue sources, cell types, and even individual cells. The most direct way to ensure the consistent quality of MSC-based therapy for cartilage regeneration is to always select the best seed cells. First, since the potential of MSCs for proliferation and chondrogenic differentiation decrease with donor age, it is essential for researchers to choose young donors to provide eligible MSCs whenever possible. Second, among various tissue-derived MSCs, WJMSCs have shown the greatest prospects for application in cartilage regeneration due to their desirable capability for proliferation, chondrogenic differentiation and immunomodulation. Third, the utilization of MSC subpopulations is an effective way to further improve the therapeutic effect of MSCs. CD271^+^ MSC subpopulations are a preferable alternative based on current knowledge, and more and better MSC subpopulations may be identified in the future with further MSC research. In addition, the processes for harvesting tissue, isolating and culturing cells, and transplanting cells in vivo need to be standardized. Furthermore, it is essential to develop unified approaches to test and characterize MSCs intended for cartilage regeneration.

## Conclusions

MSCs represent a promising cell source for cartilage repair. To improve the efficiency of MSC-based strategies, a critical first step is understanding that heterogeneity is a fundamental aspect of MSCs and obtaining an overall mastery of the characteristics of and progress of research on different MSCs in cartilage regeneration. Functional MSC heterogeneity exists among donors, tissues and MSC subpopulations, resulting in differences in their potential for cartilage repair. Selecting appropriate seed cells embodies the concept of precision medicine and will help to maximize the therapeutic effect of strategies for treating cartilage damage. However, we must realize that different laboratories, isolation methods and cell culture systems can lead to inconsistent experimental results with MSCs. Therefore, we need to be cautious about the current research results and conclusions.

## Data Availability

The authors declare that the data supporting the findings of this study are available within the paper.

## References

[1] Chen S (2017). Meniscus, articular cartilage and nucleus pulposus: a comparative review of cartilage-like tissues in anatomy, development and function. Cell Tissue Res..

[2] Loeser RF (2012). Osteoarthritis: a disease of the joint as an organ. Arthritis Rheum..

[3] Seo SJ (2014). Strategies for osteochondral repair: Focus on scaffolds. J. Tissue Eng..

[4] Fibel KH (2015). State-of-the-Art management of knee osteoarthritis. World J. Clin. Cases.

[5] McCormick F (2014). Trends in the surgical treatment of articular cartilage lesions in the United States: an analysis of a large private-payer database over a period of 8 years. Arthroscopy.

[6] Rodriguez-Lozano, F. J. et al. Allogeneic bone marrow mesenchymal stem cell transplantation in tooth extractions sites ameliorates the incidence of osteonecrotic jaw-like lesions in zoledronic acid-treated rats. *J. Clin. Med*. **9**, 1649 (2020). 10.3390/jcm9061649 PMC7355877 32486396

[7] Peng Z (2017). Glyoxalase-1 overexpression reverses defective proangiogenic function of diabetic adipose-derived stem cells in streptozotocin-induced diabetic mice model of critical limb ischemia. Stem Cells Transl. Med..

[8] Kondo S (2019). Transplantation of aggregates of autologous synovial mesenchymal stem cells for treatment of cartilage defects in the femoral condyle and the femoral groove in microminipigs. Am. J. Sports Med..

[9] Grau-Vorster, M. et al. Compliance with good manufacturing practice in the assessment of immunomodulation potential of clinical grade multipotent mesenchymal stromal cells derived from Wharton’s Jelly. *Cells*. **8**, 484 (2019). 10.3390/cells8050484 PMC6562958 31117301

[10] Maheshwer, B. et al. Regenerative potential of mesenchymal stem cells for the treatment of knee osteoarthritis and chondral defects: a systematic review and meta-analysis. *Arthroscopy*. **37**, 362–378 (2020). 10.1016/j.arthro.2020.05.037 32497658

[11] Iijima H (2018). Effectiveness of mesenchymal stem cells for treating patients with knee osteoarthritis: a meta-analysis toward the establishment of effective regenerative rehabilitation. NPJ Regen. Med..

[12] Sacchetti B (2016). No identical “mesenchymal stem cells” at different times and sites: human committed progenitors of distinct origin and differentiation potential are incorporated as adventitial cells in microvessels. Stem Cell Rep..

[13] Kalson NS (2010). Current strategies for knee cartilage repair. Int. J. Clin. Pr..

[14] Baraniak PR, McDevitt TC (2010). Stem cell paracrine actions and tissue regeneration. Regen. Med..

[15] Pan H (2020). Mesenchymal stem cells combined with tissue fusion technology promoted wound healing in porcine bowel anastomosis. Stem Cells Int..

[16] Sasaki A (2018). Canine mesenchymal stem cells from synovium have a higher chondrogenic potential than those from infrapatellar fat pad, adipose tissue, and bone marrow. PLoS ONE.

[17] Meirelles Lda S (2009). Mechanisms involved in the therapeutic properties of mesenchymal stem cells. Cytokine Growth Factor Rev..

[18] Toh WS (2014). Advances in mesenchymal stem cell-based strategies for cartilage repair and regeneration. Stem Cell Rev. Rep..

[19] Maumus M (2013). Adipose mesenchymal stem cells protect chondrocytes from degeneration associated with osteoarthritis. Stem Cell Res..

[20] Le Blanc K (2003). Immunomodulatory effects of fetal and adult mesenchymal stem cells. Cytotherapy.

[21] Gu YZ (2013). Different roles of PD-L1 and FasL in immunomodulation mediated by human placenta-derived mesenchymal stem cells. Hum. Immunol..

[22] Quaedackers ME (2009). Cell contact interaction between adipose-derived stromal cells and allo-activated T lymphocytes. Eur. J. Immunol..

[23] Kim DS (2018). Enhanced immunosuppressive properties of human mesenchymal stem cells primed by interferon-gamma. EBioMedicine.

[24] Francois M (2012). Human MSC suppression correlates with cytokine induction of indoleamine 2,3-dioxygenase and bystander M2 macrophage differentiation. Mol. Ther..

[25] Bai M (2018). IL-17A improves the efficacy of mesenchymal stem cells in ischemic-reperfusion renal injury by increasing Treg percentages by the COX-2/PGE2 pathway. Kidney Int..

[26] Petri RM (2017). Activated tissue-resident mesenchymal stromal cells regulate natural killer cell immune and tissue-regenerative function. Stem Cell Rep..

[27] Su J (2014). Phylogenetic distinction of iNOS and IDO function in mesenchymal stem cell-mediated immunosuppression in mammalian species. Cell Death Differ..

[28] Rizzo R (2008). A functional role for soluble HLA-G antigens in immune modulation mediated by mesenchymal stromal cells. Cytotherapy.

[29] Peruzzaro ST (2019). Transplantation of mesenchymal stem cells genetically engineered to overexpress interleukin-10 promotes alternative inflammatory response in rat model of traumatic brain injury. J. Neuroinflammation.

[30] McLeod CM, Mauck RL (2017). On the origin and impact of mesenchymal stem cell heterogeneity: new insights and emerging tools for single cell analysis. Eur. Cell Mater..

[31] Phinney DG (2012). Functional heterogeneity of mesenchymal stem cells: implications for cell therapy. J. Cell Biochem..

[32] Wilson A (2019). Nomenclature and heterogeneity: consequences for the use of mesenchymal stem cells in regenerative medicine. Regen. Med..

[33] Volk SW (2012). Effects of donor characteristics and ex vivo expansion on canine mesenchymal stem cell properties: implications for MSC-based therapies. Cell Transpl..

[34] Siegel G (2013). Phenotype, donor age and gender affect function of human bone marrow-derived mesenchymal stromal cells. BMC Med.

[35] Fulber J (2016). Comparative study of equine mesenchymal stem cells from healthy and injured synovial tissues: an in vitro assessment. Stem Cell Res. Ther..

[36] Trivedi A (2019). Bone marrow donor selection and characterization of MSCs is critical for pre-clinical and clinical cell dose production. J. Transl. Med..

[37] Camernik K (2020). Comprehensive analysis of skeletal muscle- and bone-derived mesenchymal stem/stromal cells in patients with osteoarthritis and femoral neck fracture. Stem Cell Res. Ther..

[38] Vinardell T (2012). A comparison of the functionality and in vivo phenotypic stability of cartilaginous tissues engineered from different stem cell sources. Tissue Eng. Part A.

[39] Lotfy A (2014). Characterization of mesenchymal stem cells derived from rat bone marrow and adipose tissue: a comparative study. Int. J. Stem Cells.

[40] Zarychta-Wisniewska W (2019). The influence of cell source and donor age on the tenogenic potential and chemokine secretion of human mesenchymal stromal cells. Stem Cells Int..

[41] La Rocca G (2013). Human Wharton’s jelly mesenchymal stem cells maintain the expression of key immunomodulatory molecules when subjected to osteogenic, adipogenic and chondrogenic differentiation in vitro: new perspectives for cellular therapy. Curr. Stem Cell Res. Ther..

[42] Dominici M (2006). Minimal criteria for defining multipotent mesenchymal stromal cells. The International Society for Cellular Therapy position statement. Cytotherapy.

[43] Phinney DG (2007). Biochemical heterogeneity of mesenchymal stem cell populations: clues to their therapeutic efficacy. Cell Cycle.

[44] Du W (2016). VCAM-1^+^ placenta chorionic villi-derived mesenchymal stem cells display potent pro-angiogenic activity. Stem Cell Res. Ther..

[45] Slukvin II, Vodyanik M (2011). Endothelial origin of mesenchymal stem cells. Cell Cycle.

[46] Liu X (2019). Heterogeneity of MSC: origin, molecular identities, and functionality. Stem Cells Int..

[47] Vasandan AB (2014). Functional differences in mesenchymal stromal cells from human dental pulp and periodontal ligament. J. Cell Mol. Med..

[48] Taguchi T (2019). Influence of donor’s age on immunomodulatory properties of canine adipose tissue-derived mesenchymal stem cells. Stem Cells Dev..

[49] Alt EU (2012). Aging alters tissue resident mesenchymal stem cell properties. Stem Cell Res.

[50] Kanawa M (2013). Age-dependent decrease in the chondrogenic potential of human bone marrow mesenchymal stromal cells expanded with fibroblast growth factor-2. Cytotherapy.

[51] Rauscher FM (2003). Aging, progenitor cell exhaustion, and atherosclerosis. Circulation.

[52] Bruna F (2016). Regenerative potential of mesenchymal stromal cells: age-related changes. Stem Cells Int.

[53] Oreffo RO (1998). Skeletal progenitor cells and ageing human populations. Clin. Sci. (Lond.).

[54] Murphy JM (2002). Reduced chondrogenic and adipogenic activity of mesenchymal stem cells from patients with advanced osteoarthritis. Arthritis Rheum..

[55] Dudics V (2009). Chondrogenic potential of mesenchymal stem cells from patients with rheumatoid arthritis and osteoarthritis: measurements in a microculture system. Cells Tissues Organs.

[56] Scharstuhl A (2007). Chondrogenic potential of human adult mesenchymal stem cells is independent of age or osteoarthritis etiology. Stem Cells.

[57] Badraiq H (2017). Effects of maternal obesity on Wharton’s Jelly mesenchymal stromal cells. Sci. Rep..

[58] Herrmann, M. et al. Phenotypic characterization of bone marrow mononuclear cells and derived stromal cell populations from human iliac crest, vertebral body and femoral Head. *Int. J. Mol. Sci*. **20**, 3454 (2019). 10.3390/ijms20143454 PMC6678175 31337109

[59] Bornes TD (2018). Articular cartilage repair with mesenchymal stem cells after chondrogenic priming: a pilot study. Tissue Eng. Part A.

[60] Sun Y (2018). Citrullinated fibrinogen impairs immunomodulatory function of bone marrow mesenchymal stem cells by triggering toll-like receptor. Clin. Immunol..

[61] Ding J (2016). Bone marrow mesenchymal stem cell-based engineered cartilage ameliorates polyglycolic acid/polylactic acid scaffold-induced inflammation through m2 polarization of macrophages in a pig model. Stem Cells Transl. Med..

[62] Jin L (2019). Osteochondral tissue regenerated via a strategy by stacking pre-differentiated BMSC sheet on fibrous mesh in a gradient. Biomed. Mater..

[63] Sun Y (2019). 3D-bioprinting a genetically inspired cartilage scaffold with GDF5-conjugated BMSC-laden hydrogel and polymer for cartilage repair. Theranostics.

[64] He A (2017). Repair of osteochondral defects with in vitro engineered cartilage based on autologous bone marrow stromal cells in a swine model. Sci. Rep..

[65] Orozco L (2013). Treatment of knee osteoarthritis with autologous mesenchymal stem cells: a pilot study. Transplantation.

[66] Soler R (2016). Final results of a phase I-II trial using ex vivo expanded autologous Mesenchymal Stromal Cells for the treatment of osteoarthritis of the knee confirming safety and suggesting cartilage regeneration. Knee.

[67] Chahal J (2019). Bone marrow mesenchymal stromal cell treatment in patients with osteoarthritis results in overall improvement in pain and symptoms and reduces synovial inflammation. Stem Cells Transl. Med..

[68] Teo AQA (2019). Equivalent 10-year outcomes after implantation of autologous bone marrow-derived mesenchymal stem cells versus autologous chondrocyte implantation for chondral defects of the knee. Am. J. Sports Med..

[69] Hashimoto Y (2019). Transplantation of autologous bone marrow-derived mesenchymal stem cells under arthroscopic surgery with microfracture versus microfracture alone for articular cartilage lesions in the knee: a multicenter prospective randomized control clinical trial. Regen. Ther..

[70] Vega A (2015). Treatment of knee osteoarthritis with allogeneic bone marrow mesenchymal stem cells: a randomized controlled trial. Transplantation.

[71] Wu SC (2018). Hyaluronan microenvironment enhances cartilage regeneration of human adipose-derived stem cells in a chondral defect model. Int. J. Biol. Macromol..

[72] Mei L (2017). Culture-expanded allogenic adipose tissue-derived stem cells attenuate cartilage degeneration in an experimental rat osteoarthritis model. PLoS ONE.

[73] Dragoo JL, Chang W (2017). Arthroscopic harvest of adipose-derived mesenchymal stem cells from the infrapatellar fat pad. Am. J. Sports Med..

[74] Winter A (2003). Cartilage-like gene expression in differentiated human stem cell spheroids: a comparison of bone marrow-derived and adipose tissue-derived stromal cells. Arthritis Rheum..

[75] Kim HJ, Im GI (2009). Chondrogenic differentiation of adipose tissue-derived mesenchymal stem cells: greater doses of growth factor are necessary. J. Orthop. Res..

[76] Danisovic L (2009). Comparison of in vitro chondrogenic potential of human mesenchymal stem cells derived from bone marrow and adipose tissue. Gen. Physiol. Biophys..

[77] Vidal MA (2012). Evaluation of senescence in mesenchymal stem cells isolated from equine bone marrow, adipose tissue, and umbilical cord tissue. Stem Cells Dev..

[78] Li CY (2015). Comparative analysis of human mesenchymal stem cells from bone marrow and adipose tissue under xeno-free conditions for cell therapy. Stem Cell Res. Ther..

[79] Ueyama H (2020). Local transplantation of adipose-derived stem cells has a significant therapeutic effect in a mouse model of rheumatoid arthritis. Sci. Rep..

[80] Oshima T (2019). A scaffold-free allogeneic construct from adipose-derived stem cells regenerates an osteochondral defect in a rabbit model. Arthroscopy.

[81] Dubey NK (2019). Adipose-derived stem cells attenuates diabetic osteoarthritis via inhibition of glycation-mediated inflammatory cascade. Aging Dis..

[82] Oh SJ (2020). Comparative analysis of adipose-derived stromal cells and their secretome for auricular cartilage regeneration. Stem Cells Int..

[83] Ishihara M (2018). Biomaterials as cell carriers for augmentation of adipose tissue-derived stromal cell transplantation. Biomed. Mater. Eng..

[84] Kurzyk A (2019). Characterization and optimization of the seeding process of adipose stem cells on the polycaprolactone scaffolds. Stem Cells Int..

[85] Boyer C (2020). A self-setting hydrogel of silylated chitosan and cellulose for the repair of osteochondral defects: from in vitro characterization to preclinical evaluation in dogs. Front. Bioeng. Biotechnol..

[86] Lee WS (2019). Intra-articular injection of autologous adipose tissue-derived mesenchymal stem cells for the treatment of knee osteoarthritis: a phase IIb, randomized, placebo-controlled clinical trial. Stem Cells Transl. Med..

[87] Kim YS, Koh YG (2018). Comparative matched-pair analysis of open-wedge high tibial osteotomy with versus without an injection of adipose-derived mesenchymal stem cells for varus knee osteoarthritis: clinical and second-look arthroscopic results. Am. J. Sports Med..

[88] Pers YM (2018). Injection of adipose-derived stromal cells in the knee of patients with severe osteoarthritis has a systemic effect and promotes an anti-inflammatory phenotype of circulating immune cells. Theranostics.

[89] Pers YM (2016). Adipose mesenchymal stromal cell-based therapy for severe osteoarthritis of the knee: a phase I dose-escalation trial. Stem Cells Transl. Med..

[90] Jo CH (2014). Intra-articular injection of mesenchymal stem cells for the treatment of osteoarthritis of the knee: a proof-of-concept clinical trial. Stem Cells.

[91] Song Y (2018). Human adipose-derived mesenchymal stem cells for osteoarthritis: a pilot study with long-term follow-up and repeated injections. Regen. Med..

[92] De Bari C (2001). Multipotent mesenchymal stem cells from adult human synovial membrane. Arthritis Rheum..

[93] Ferro T (2019). Successful isolation and ex vivo expansion of human mesenchymal stem/stromal cells obtained from different synovial tissue-derived (biopsy) samples. J. Cell Physiol..

[94] Amemiya M (2020). Synovial fluid-derived mesenchymal cells have non-inferior chondrogenic potential and can be utilized for regenerative therapy as substitute for synovium-derived cells. Biochem. Biophys. Res. Commun..

[95] Zheng YL (2015). Mesenchymal stem cells obtained from synovial fluid mesenchymal stem cell-derived induced pluripotent stem cells on a matrigel coating exhibited enhanced proliferation and differentiation potential. PLoS ONE.

[96] Yoshimura H (2007). Comparison of rat mesenchymal stem cells derived from bone marrow, synovium, periosteum, adipose tissue, and muscle. Cell Tissue Res..

[97] Gale AL (2019). Comparison of the chondrogenic differentiation potential of equine synovial membrane-derived and bone marrow-derived mesenchymal stem cells. Front. Vet. Sci..

[98] Djouad F (2005). Transcriptional profiles discriminate bone marrow-derived and synovium-derived mesenchymal stem cells. Arthritis Res. Ther..

[99] Kim TW (2018). Direct coculture of human chondrocytes and synovium-derived stem cells enhances in vitro chondrogenesis. Cell J..

[100] Pei M, He F (2012). Extracellular matrix deposited by synovium-derived stem cells delays replicative senescent chondrocyte dedifferentiation and enhances redifferentiation. J. Cell Physiol..

[101] Koga H (2007). Synovial stem cells are regionally specified according to local microenvironments after implantation for cartilage regeneration. Stem Cells.

[102] Lee JC (2012). Synovium-derived mesenchymal stem cells encapsulated in a novel injectable gel can repair osteochondral defects in a rabbit model. Tissue Eng. Part A.

[103] Sekiya I (2015). Arthroscopic transplantation of synovial stem cells improves clinical outcomes in knees with cartilage defects. Clin. Orthop. Relat. Res..

[104] Akgun I (2015). Matrix-induced autologous mesenchymal stem cell implantation versus matrix-induced autologous chondrocyte implantation in the treatment of chondral defects of the knee: a 2-year randomized study. Arch. Orthop. Trauma Surg..

[105] McElreavey KD (1991). Isolation, culture and characterisation of fibroblast-like cells derived from the Wharton’s jelly portion of human umbilical cord. Biochem. Soc. Trans..

[106] Fong CY (2011). Human Wharton’s jelly stem cells have unique transcriptome profiles compared to human embryonic stem cells and other mesenchymal stem cells. Stem Cell Rev. Rep..

[107] Donders R (2018). Human Wharton’s jelly-derived stem cells display a distinct immunomodulatory and proregenerative transcriptional signature compared to bone marrow-derived stem cells. Stem Cells Dev..

[108] Wu KH (2013). Human application of ex vivo expanded umbilical cord-derived mesenchymal stem cells: enhance hematopoiesis after cord blood transplantation. Cell Transpl..

[109] Reppel L (2015). Chondrogenic induction of mesenchymal stromal/stem cells from Wharton’s jelly embedded in alginate hydrogel and without added growth factor: an alternative stem cell source for cartilage tissue engineering. Stem Cell Res. Ther..

[110] Wang L (2009). A comparison of human bone marrow-derived mesenchymal stem cells and human umbilical cord-derived mesenchymal stromal cells for cartilage tissue engineering. Tissue Eng. Part A.

[111] Weiss ML (2008). Immune properties of human umbilical cord Wharton’s jelly-derived cells. Stem Cells.

[112] Liu S (2012). Immune characterization of mesenchymal stem cells in human umbilical cord Wharton’s jelly and derived cartilage cells. Cell Immunol..

[113] Zhou C (2011). Immunomodulatory effect of human umbilical cord Wharton’s jelly-derived mesenchymal stem cells on lymphocytes. Cell Immunol..

[114] Saulnier N (2015). Intra-articular administration of xenogeneic neonatal Mesenchymal Stromal Cells early after meniscal injury down-regulates metalloproteinase gene expression in synovium and prevents cartilage degradation in a rabbit model of osteoarthritis. Osteoarthr. Cartil..

[115] Cheng JH (2019). Comparison efficacy of ESWT and Wharton’s jelly mesenchymal stem cell in early osteoarthritis of rat knee. Am. J. Transl. Res..

[116] Zhang Y (2020). Co-culture of hWJMSCs and pACs in double biomimetic ACECM oriented scaffold enhances mechanical properties and accelerates articular cartilage regeneration in a caprine model. Stem Cell Res. Ther..

[117] Gao LR (2015). Intracoronary infusion of Wharton’s jelly-derived mesenchymal stem cells in acute myocardial infarction: double-blind, randomized controlled trial. BMC Med..

[118] Hu J (2013). Long term effects of the implantation of Wharton’s jelly-derived mesenchymal stem cells from the umbilical cord for newly-onset type 1 diabetes mellitus. Endocr. J..

[119] Sadlik B (2017). Knee Cartilage Regeneration with Umbilical Cord Mesenchymal Stem Cells Embedded in Collagen Scaffold Using Dry Arthroscopy Technique. Adv. Exp. Med. Biol.

[120] Kudva AK (2018). RGD-functionalized polyethylene glycol hydrogels support proliferation and in vitro chondrogenesis of human periosteum-derived cells. J. Biomed. Mater. Res. A.

[121] Nogami M (2012). Isolation and characterization of human amniotic mesenchymal stem cells and their chondrogenic differentiation. Transplantation.

[122] Spaas JH (2015). Chondrogenic priming at reduced cell density enhances cartilage adhesion of equine allogeneic MSCs-a loading sensitive phenomenon in an organ culture study with 180 explants. Cell Physiol. Biochem..

[123] Riekstina U (2009). Embryonic stem cell marker expression pattern in human mesenchymal stem cells derived from bone marrow, adipose tissue, heart and dermis. Stem Cell Rev. Rep..

[124] Thomson TM (1988). Expression of human nerve growth factor receptor on cells derived from all three germ layers. Exp. Cell Res..

[125] Barilani M (2018). Low-affinity nerve growth factor receptor (CD271) heterogeneous expression in adult and fetal mesenchymal stromal cells. Sci. Rep..

[126] Hermida-Gomez T (2011). Bone marrow cells immunomagnetically selected for CD271^+^ antigen promote in vitro the repair of articular cartilage defects. Tissue Eng. Part A.

[127] Quirici N (2010). Anti-L-NGFR and -CD34 monoclonal antibodies identify multipotent mesenchymal stem cells in human adipose tissue. Stem Cells Dev..

[128] Jarocha D (2008). Adventage of mesenchymal stem cells (MSC) expansion directly from purified bone marrow CD105^+^ and CD271^+^ cells. Folia Histochem. Cytobiol..

[129] Quirici N (2002). Isolation of bone marrow mesenchymal stem cells by anti-nerve growth factor receptor antibodies. Exp. Hematol..

[130] Mifune Y (2013). Therapeutic superiority for cartilage repair by CD271-positive marrow stromal cell transplantation. Cell Transpl..

[131] Kohli N (2019). CD271-selected mesenchymal stem cells from adipose tissue enhance cartilage repair and are less angiogenic than plastic adherent mesenchymal stem cells. Sci. Rep..

[132] Kuci S (2010). CD271 antigen defines a subset of multipotent stromal cells with immunosuppressive and lymphohematopoietic engraftment-promoting properties. Haematologica.

[133] Kuci Z (2013). Clonal analysis of multipotent stromal cells derived from CD271^+^ bone marrow mononuclear cells: functional heterogeneity and different mechanisms of allosuppression. Haematologica.

[134] Arufe MC (2010). Chondrogenic potential of subpopulations of cells expressing mesenchymal stem cell markers derived from human synovial membranes. J. Cell Biochem..

[135] Rodin S (2014). Clonal culturing of human embryonic stem cells on laminin-521/E-cadherin matrix in defined and xeno-free environment. Nat. Commun..

[136] Huntsman HD (2015). Human hematopoietic stem cells from mobilized peripheral blood can be purified based on CD49f integrin expression. Blood.

[137] Rosa AI (2016). Heterocellular contacts with mouse brain endothelial cells via laminin and alpha6beta1 integrin sustain subventricular zone (SVZ) stem/progenitor cells properties. Front. Cell Neurosci..

[138] Dall GV (2017). SCA-1 labels a subset of estrogen-responsive bipotential repopulating cells within the CD24(^+^) CD49f(hi) mammary stem cell-enriched compartment. Stem Cell Rep..

[139] Toya SP (2015). Integrin alpha6beta1 expressed in ESCs instructs the differentiation to endothelial cells. Stem Cells.

[140] Bigoni-Ordonez GD (2019). Integrin alpha6 (CD49f), the microenvironment and cancer stem cells. Curr. Stem Cell Res. Ther..

[141] Ouji Y (2010). Effects of Wnt-10b on proliferation and differentiation of adult murine skin-derived CD34 and CD49f double-positive cells. J. Biosci. Bioeng..

[142] Krebsbach PH, Villa-Diaz LG (2017). The role of integrin alpha6 (CD49f) in stem cells: more than a conserved biomarker. Stem Cells Dev..

[143] Hakki SS (2015). Comparison of mesenchymal stem cells isolated from pulp and periodontal ligament. J. Periodontol..

[144] Yang Z (2015). CD49f acts as an inflammation sensor to regulate differentiation, adhesion, and migration of human mesenchymal stem cells. Stem Cells.

[145] Yu KR (2012). CD49f enhances multipotency and maintains stemness through the direct regulation of OCT4 and SOX2. Stem Cells.

[146] Marecic O (2015). Identification and characterization of an injury-induced skeletal progenitor. Proc. Natl Acad. Sci. USA.

[147] Garcia-Alvarez F (2011). Chondrogenic differentiation in femoral bone marrow-derived mesenchymal cells (MSC) from elderly patients suffering osteoarthritis or femoral fracture. Arch. Gerontol. Geriatr..

[148] Covas DT (2008). Multipotent mesenchymal stromal cells obtained from diverse human tissues share functional properties and gene-expression profile with CD146^+^ perivascular cells and fibroblasts. Exp. Hematol..

[149] Paduano F (2016). CD146 expression influences periapical cyst mesenchymal stem cell properties. Stem Cell Rev. Rep..

[150] Espagnolle N (2014). CD146 expression on mesenchymal stem cells is associated with their vascular smooth muscle commitment. J. Cell Mol. Med.

[151] Zhu W (2013). Comparison of the properties of human CD146^+^ and CD146^−^ periodontal ligament cells in response to stimulation with tumour necrosis factor alpha. Arch. Oral. Biol..

[152] Xu J (2009). Multiple differentiation capacity of STRO-1^+^/CD146^+^ PDL mesenchymal progenitor cells. Stem Cells Dev..

[153] Tsang WP (2013). CD146^+^ human umbilical cord perivascular cells maintain stemness under hypoxia and as a cell source for skeletal regeneration. PLoS ONE.

[154] Kouroupis D (2014). The assessment of CD146-based cell sorting and telomere length analysis for establishing the identity of mesenchymal stem cells in human umbilical cord. F1000Res..

[155] Hagmann S (2014). Fluorescence activated enrichment of CD146^+^ cells during expansion of human bone-marrow derived mesenchymal stromal cells augments proliferation and GAG/DNA content in chondrogenic media. BMC Musculoskelet. Disord..

[156] Anfosso F (2001). Outside-in signaling pathway linked to CD146 engagement in human endothelial cells. J. Biol. Chem..

[157] Kara N (2017). miR-27 regulates chondrogenesis by suppressing focal adhesion kinase during pharyngeal arch development. Dev. Biol..

[158] Wu CC (2016). CD146^+^ mesenchymal stem cells display greater therapeutic potential than CD146- cells for treating collagen-induced arthritis in mice. Stem Cell Res. Ther..

[159] Li X (2019). Enrichment of CD146(^+^) adipose-derived stem cells in combination with articular cartilage extracellular matrix scaffold promotes cartilage regeneration. Theranostics.

[160] Hindle P (2016). Perivascular mesenchymal stem cells in sheep: characterization and autologous transplantation in a model of articular cartilage repair. Stem Cells Dev..

[161] Barry FP (1999). The monoclonal antibody SH-2, raised against human mesenchymal stem cells, recognizes an epitope on endoglin (CD105). Biochem. Biophys. Res. Commun..

[162] Consentius C (2018). In situ detection of CD73^+^ CD90^+^ CD105^+^ lineage: mesenchymal stromal cells in human placenta and bone marrow specimens by chipcytometry. Cytom. A.

[163] Spiropoulos A (2011). Rapid clinical-scale propagation of mesenchymal stem cells using cultures initiated with immunoselected bone marrow CD105^+^ cells. J. Cell Mol. Med..

[164] Fan W (2016). CD105 promotes chondrogenesis of synovium-derived mesenchymal stem cells through Smad2 signaling. Biochem. Biophys. Res. Commun..

[165] Chang CB (2013). Chondrogenic potentials of human synovium-derived cells sorted by specific surface markers. Osteoarthr. Cartil..

[166] Qi J (2011). Proliferation and chondrogenic differentiation of CD105-positive enriched rat synovium-derived mesenchymal stem cells in three-dimensional porous scaffolds. Biomed. Mater..

[167] Anderson P (2013). CD105 (endoglin)-negative murine mesenchymal stromal cells define a new multipotent subpopulation with distinct differentiation and immunomodulatory capacities. PLoS ONE.

[168] Stewart K (1999). Further characterization of cells expressing STRO-1 in cultures of adult human bone marrow stromal cells. J. Bone Min. Res..

[169] Lin G (2011). Tissue distribution of mesenchymal stem cell marker Stro-1. Stem Cells Dev..

[170] Koyama N (2011). Pluripotency of mesenchymal cells derived from synovial fluid in patients with temporomandibular joint disorder. Life Sci..

[171] Ranga Rao S, Subbarayan R (2019). Passage-dependent expression of STRO-1 in human gingival mesenchymal stem cells. J. Cell Biochem..

[172] Psaltis PJ (2010). Enrichment for STRO-1 expression enhances the cardiovascular paracrine activity of human bone marrow-derived mesenchymal cell populations. J. Cell Physiol..

[173] Gothard D (2014). Prospective isolation of human bone marrow stromal cell subsets: A comparative study between Stro-1-, CD146- and CD105-enriched populations. J. Tissue Eng..

[174] Wen L, Tang F (2016). Single-cell sequencing in stem cell biology. Genome Biol..

[175] Yin Z (2016). Single-cell analysis reveals a nestin(^+^) tendon stem/progenitor cell population with strong tenogenic potentiality. Sci. Adv..

[176] Freeman BT (2015). Single-Cell RNA-seq of bone marrow-derived mesenchymal stem cells reveals unique profiles of lineage priming. PLoS ONE.

[177] Tikhonova AN (2019). The bone marrow microenvironment at single-cell resolution. Nature.

[178] Baryawno N (2019). A cellular taxonomy of the bone marrow stroma in homeostasis and leukemia. Cell.

[179] Barrett AN (2019). Human Wharton’s jelly mesenchymal stem cells show unique gene expression compared with bone marrow mesenchymal stem cells using single-cell RNA-sequencing. Stem Cells Dev..

[180] Wang, Z. et al. Single-cell RNA sequencing deconvolutes the in vivo heterogeneity of human bone marrow-derived mesenchymal stem cells. Preprint at https://www.biorxiv.org/content/10.1101/2020.04.06.027904v2.full (2020) 10.7150/ijbs.61950 PMC8579438 34803492

[181] Konig, I. R. et al. What is precision medicine? *Eur. Respir. J*. **50**, 1700391 (2017). 10.1183/13993003.00391-2017 29051268

[182] Yu Y (2019). PreMedKB: an integrated precision medicine knowledgebase for interpreting relationships between diseases, genes, variants and drugs. Nucleic Acids Res.

[183] Roelofs AJ (2017). Joint morphogenetic cells in the adult mammalian synovium. Nat. Commun..

